# One Size Does not Fit All: Gamified Escape Room Learning for Students With ADHD and Language Difficulties in a Quasi‐Experimental Cohort Study

**DOI:** 10.1002/nop2.70882

**Published:** 2026-09-30

**Authors:** Dorit Weil‐Lotan, Revital Harari, Carolina Tannenbaum‐Baruchi, Miriam Theilla

**Affiliations:** ^1^ School of Nursing Sciences The Academic College of Tel Aviv–Yaffo Tel Aviv–Yaffo Israel

**Keywords:** attention deficit disorder with hyperactivity, language disorders, nursing education, nursing students, teaching methods

## Abstract

**Aim:**

This study assessed the association between digital escape room‐based instruction and academic performance among nursing students, with particular attention to students with attention‐deficit hyperactivity disorder (ADHD) and language‐related challenges.

**Design:**

A retrospective quasi‐experimental study compared the effects of digital escape room‐based instruction with traditional teaching methods. Students enrolled in the 2024 and 2025 academic years received escape room instruction (intervention group), whereas students enrolled in the 2023 academic year received traditional instruction (control group). The course was taught by the same instructor throughout.

**Methods:**

Academic performance data from 426 nursing students were analysed (intervention: *n* = 276; control: *n* = 150). Two‐way analyses of covariance (ANCOVAs) examined the interaction between instructional condition and learner status (no difficulties, language difficulties or learning difficulties/ADHD), with age and gender as covariates. Outcomes included multiple‐choice (MC), constructed response (CR) and total exam scores, with post hoc comparisons corrected using the Holm procedure.

**Results:**

The Escape Room × Learner Status interaction showed significant but modest interaction effects for all outcomes: CR scores (*F*(2, 366) = 3.01, *p* = 0.05, *η* ^2^ *p* = 0.02), MC scores (*F*(2, 366) = 6.11, *p* < 0.01, *η* ^2^ *p* = 0.03) and total scores (*F*(2, 366) = 5.45, *p* < 0.01, *η* ^2^ *p* = 0.03). Students with language difficulties scored significantly lower on CR (*d* = −0.63, 95% CI [−0.97, −0.29]) and total scores (*d* = −0.57, 95% CI [−0.90, −0.23]) under escape room instruction. Students with learning difficulties/ADHD showed higher MC scores under escape room instruction (*d* = 0.68, 95% CI [0.17, 1.18]).

**Conclusion:**

The findings suggest that gamified escape room instruction may have differential effects on academic performance across learner groups. Students with learning difficulties/ADHD demonstrated better performance on recognition‐based tasks, whereas students with language difficulties may face challenges, particularly on tasks requiring written responses. In the context of nursing education, it is crucial to assess and adapt gamified teaching strategies to meet different learning needs rather than relying on a uniform approach. Nursing educators are encouraged to consider combining gamified learning with other teaching and assessment methods to promote inclusive and student‐centered learning environments. When implementing gamified techniques, it is essential in nursing practice to tailor educational approaches to the varied learning needs of nursing students, particularly those with ADHD and other learning challenges. Multicenter research is needed to confirm these findings.

**Patient or Public Contribution:**

This educational research study did not involve the participation of patients or the public in its design, execution or reporting.

## 1 Introduction

Nursing curricula present significant challenges for students, demanding comprehensive mastery of foundational theoretical knowledge across multiple disciplines including anatomy, physiology, pharmacology and biochemistry. These subjects form the cornerstone for effective patient assessment, safe medication administration and competent clinical interventions (Corlett et al. 2003). The primary objective of nursing education is to establish a robust foundation for safe and effective nursing practice while facilitating the critical translation of theoretical knowledge into practical, patient‐centered care (Casey et al. 2011). The quality of this educational foundation has lasting implications, as structured support during the transition from education to practice has been shown to improve retention, professional confidence and clinical competence among new graduate nurses (Ulrich et al. 2010). The unique pressures facing nursing students stem from the need to balance academic rigour with the demanding nature of clinical practice, with sources of stress varying across years of study and relating to both academic workload and clinical experiences (Lavoie‐Tremblay et al. 2022).

The substantial volume of information, coupled with the imperative for practical application, can overwhelm students and necessitate considerable effort as well as effective study strategies (Ghasemi et al. 2020). Within this context, Bloom's Taxonomy provides a valuable framework for assessing different levels of cognitive skills, ranging from basic recall to advanced higher‐order thinking (Su and Osisek 2011).

The increasing prevalence of nursing students diagnosed with attention‐deficit hyperactivity disorder (ADHD) is primarily due to enhanced recognition and reduced stigma surrounding the condition (Kooij et al. 2019). According to the World Health Organization (WHO 2019), ADHD is a neurodevelopmental disorder that begins in childhood and often persists into adulthood, characterized by clinical symptoms of inattention and/or hyperactivity–impulsivity that cause functional impairments in multiple settings, including home, education, work or social relationships. Consequently, growing numbers of nursing students are being identified with ADHD (Kallivayalil 2012). These students frequently encounter distinctive challenges that may adversely affect their academic performance (Gormley et al. 2018). The symptoms of ADHD can contribute to heightened stress, anxiety and burnout and present significant obstacles to success in nursing education (Liepinaitiene et al. 2024).

Virtual escape rooms represent an innovative pedagogical approach where participants solve puzzles and complete tasks to ‘escape’ a virtual environment, often within a set time limit (Asad et al. 2021). Research suggests that gamified activities can enhance attention, task persistence and academic outcomes for students with ADHD (Legaki et al. 2020; DuPaul et al. 1998). However, the evidence regarding the effectiveness of gamification in education is mixed and context dependent. Although some studies report benefits including increased engagement and improved learning outcomes (Grech and Grech 2021; Borrego et al. 2017), others have found that the effects vary substantially depending on the specific population, learning objectives and implementation characteristics (Kim et al. 2024; Pozo‐Sanchez et al. 2022). Notably, Guckian et al. (2020) cautioned that the effectiveness of escape rooms for all learners, including those with ADHD, remains uncertain.

The growing globalization of healthcare presents additional challenges for nursing students, particularly regarding language proficiency (Crawford and Candlin 2013). Language barriers can impede student engagement in class discussions and comprehension of complex medical terminology, with the potential for increased stress and diminished mental well‐being (Zhao et al. 2024). Students with language difficulties frequently struggle with reading comprehension, written expression and oral communication, ultimately affecting their academic performance across various subjects (Corlett et al. 2003). Instructional methods that do not align with a student's individual profile may lead to increased stress and negative outcomes. The stress encountered by students with language difficulties can weaken their professional identity and raise the risks of burnout and dropout (Zhao et al. 2024; Vabo et al. 2022; Hoeve et al. 2014). Offering supportive teaching environments and fostering positive faculty‐student relationships can help reduce this stress and strengthen students' professional socialization and commitment to the profession (Del Prato et al. 2011).

Despite the growing interest in gamified pedagogies, a notable gap persists in the literature: studies examining escape room effectiveness in nursing education have often neglected to address whether the benefits of these approaches are equitably distributed among diverse student populations (Sainz‐Rozas et al. 2025). There is limited evidence regarding whether escape room instruction differentially affects students with ADHD or language difficulties compared to students without such challenges. Given the heterogeneous needs of contemporary nursing student populations, the traditional one‐size‐fits‐all approach to evaluating educational innovations is insufficient. From a theoretical perspective, we anticipate that different groups of learners will respond differently to escape room pedagogy. The interactive and feedback‐rich nature of escape rooms is expected to increase engagement among students with ADHD or learning challenges. On the other hand, the emphasis on rapid reading, written instructions and specialized clinical language may create greater cognitive and linguistic challenges for students with language difficulties.

This study was therefore designed to address this gap by examining the following research aims:

- Compared academic performance (MC scores, CR scores and total exam scores) between students exposed to digital escape room instruction and those receiving traditional instruction.
- To examine whether instructional effects differ among students with ADHD/learning difficulties and students with language difficulties.
- To assess whether instructional format is differentially associated with performance across Bloom's Taxonomy cognitive levels.

Given the limited and mixed prior evidence, this study was primarily exploratory in nature, and directional hypotheses were not pre‐specified.

## 2 Methods

This retrospective quasi‐experimental study examined the association between a virtual escape room learning intervention and nursing students' academic performance. The research was conducted as part of a mandatory undergraduate nursing theory course at a nursing school during the 2023–2025 academic years.

Students were assigned to conditions based on their year of enrollment. The control group comprised all students enrolled in the 2023 academic year, who received traditional instruction (*n* = 150). The escape room intervention was introduced in the 2024 academic year and continued in 2025; all students enrolled in those years constituted the intervention group (*n* = 276). This allocation was naturalistic, following from the institutional decision to adopt the escape room as a pedagogical tool beginning in 2024, and was not manipulated for research purposes. The study was approved by the relevant institutional ethics committee (Approval No. 005‐24‐MTA).

### 2.1 Participants

#### 2.1.1 Inclusion Criteria

Undergraduate nursing students enrolled in the specified mandatory theoretical course during the 2023–2025 academic years who completed the final examination.

#### 2.1.2 Exclusion Criteria

Four students who submitted blank examination forms (grade = 0) were excluded, as these were interpreted as non‐attempts rather than poor performance.

The final study sample comprised 426 undergraduate nursing students. Participants' ages ranged from 18 to 54.82 years (*M* = 25.31, SD = 5.32). The sample included 304 women (80.6%) and 73 men (19.4%). Approximately half of the students (47.2%) identified as belonging to Arab society. Demographic information on age, gender and ethnicity was unavailable for 53 participants (12.3%) due to the institution's data retention policy, which removes demographic records for students who have completed their studies or formally withdrawn from the institution. Institutional inquiry confirmed that these 53 participants were distributed across all three academic years, suggesting that the missingness was related to administrative processes rather than participant characteristics, though non‐random missingness cannot be fully excluded. These cases were included in all analyses not requiring the missing variables.

Students were classified as having language difficulties (*n* = 153), learning difficulties including ADHD (*n* = 32) or no difficulties (*n* = 241). All classifications were based on formal institutional records maintained by the student support services office, reflecting officially documented diagnoses or recognized accommodations, not self‐report. Students classified with ADHD or learning/language difficulties were eligible to receive 30 min of additional examination time. The distribution of learner categories did not differ significantly between the intervention and control groups (*χ* ^2^(2) = 0.77, *p* = 0.68). The participants flow diagram is illustrated in Figure 1.

**FIGURE 1:**
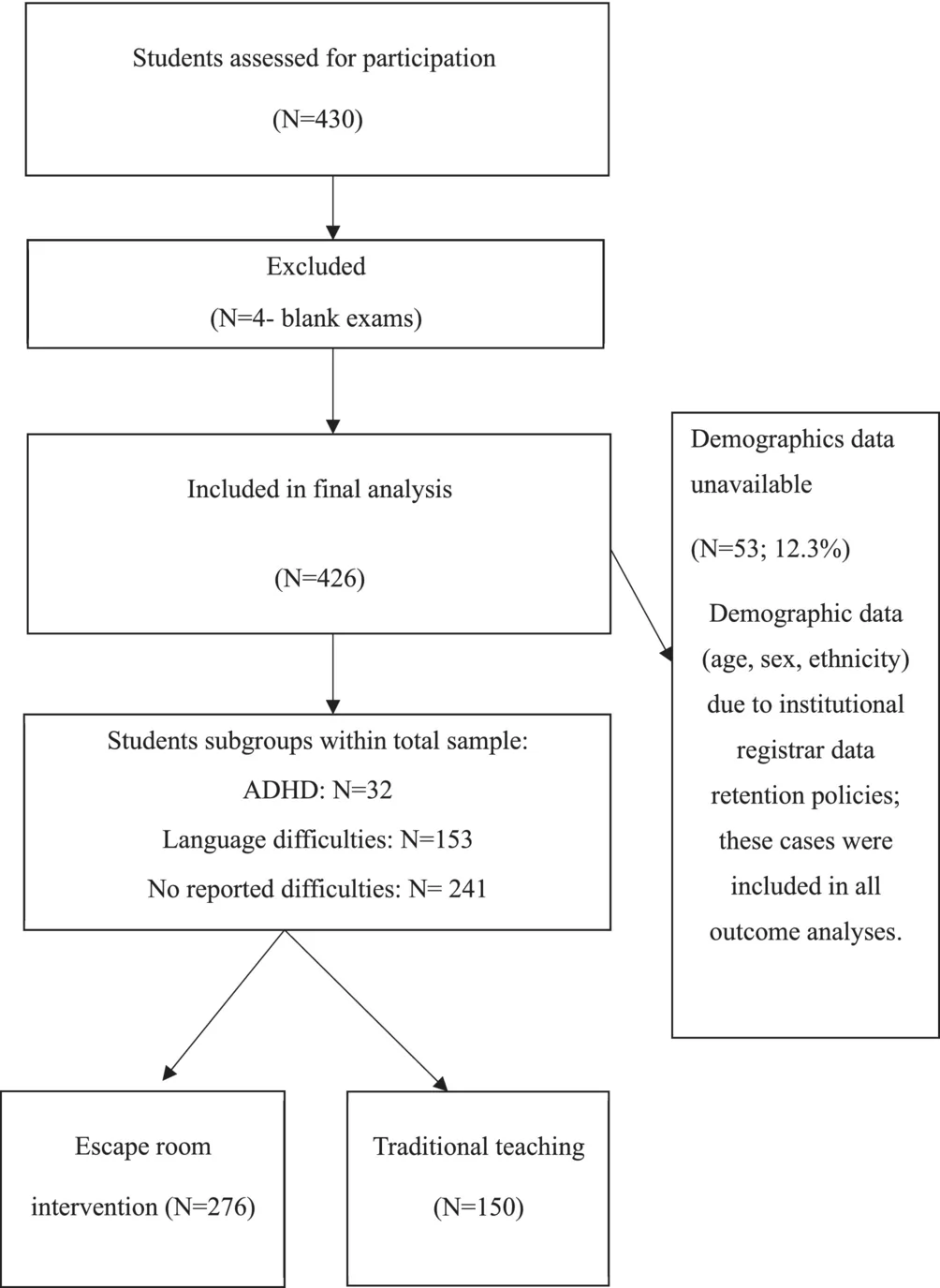
Participants flow diagram (STROBE).

### 2.2 Instructional Interventions

The virtual digital escape room activity was conducted on the Genially platform with the goal of reinforcing fundamental nursing theory concepts based on Bloom's Taxonomy. This activity was introduced as a supplementary exercise after the regular course curriculum. Students participated in the escape room activity individually using their personal computers. The activity lasted between 30 and 60 min, depending on each student's pace and knowledge level, and consisted of eight tasks. These tasks involved classifying nursing theories, matching concepts with definitions, identifying nursing theorists using clues, interpreting theoretical concepts from images, drag‐and‐drop classification exercises and answering multiple‐choice questions related to the nursing theories covered in the course. Students had to successfully complete each task to progress through the activity, solving clinical puzzles in the virtual environment to apply, analyse and synthesize course material. The activity was self‐paced and non‐competitive, allowing students unlimited attempts and the option to refer to course materials. Although the instructor was available for questions, students completed the activity independently, without structured facilitation or a debriefing session. The escape room tasks can be viewed in Appendix S1. Student participation was monitored by the same instructor through platform logs, and completion of the escape room was part of the course content review and final exam preparation.

On the other hand, the control group received the same course instruction but did not participate in the escape room activity. Both groups underwent the same final assessment.

### 2.3 Data Sources and Variables

Academic performance data (exam scores, item‐level responses) were extracted from the institution's Learning Management System (LMS) and merged with institutional records indicating ADHD/LD status using anonymized student identifiers. Data were anonymized prior to transferring to the study statistician for analysis. Anonymized data were stored on the statistician's password‐protected computer, accessible only to members of the research team. Before statistical analysis, the merged dataset was reviewed for completeness, duplicate records and consistency of anonymous identifiers. Records with missing or inconsistent identifiers were verified against the original institutional database prior to anonymization. Data cleaning was limited to the predefined exclusion criteria described above, and no additional modifications were made to the dataset before analysis.

There were three main outcomes: multiple‐choice (MC), constructed‐response (CR) and total scores (final exam score). The final exam comprised both MC and CR sections, assessing the same course content covered under both instructional conditions. Although the exams consistently assessed the same course learning objectives and drew on the same curricular content, specific questions differed year to year, as is typical of recurring university courses. The relative proportions of MC and CR items and their respective weighting in the final grade varied across years. CR responses were scored by a single grader who was blinded to the students' instructional condition. All test items were categorized according to Bloom's cognitive levels (levels 1–3 for CR questions and levels 1–2 for MC questions) by the study authors using Bloom's revised taxonomy framework. Only cognitive levels 1–3 (Remember, Understand, Apply) were identified in the exam items. Formal inter‐rater reliability testing was not conducted for this classification. All scores were presented as percentage points (0–100).

### 2.4 Statistical Analyses

All analyses used SPSS v26 and JASP. The analytic strategy proceeded in two stages. First, independent samples *t*‐tests were used for between‐group comparisons on each outcome measure, with significance set at *p* < 0.05 and effect sizes reported using Cohen's *d* with 95% confidence intervals. Welch's correction was applied where Levene's test indicated unequal variances.

Second, to formally test whether the effect of instructional condition differed across learner subgroups, two‐way analyses of covariance (ANCOVAs) were conducted for each outcome, with instructional condition (Escape Room vs. Traditional) and learner status (No difficulties, Language difficulties, Learning difficulties/ADHD) as fixed factors and age and gender as covariates. Post hoc pairwise comparisons were corrected using the Holm procedure to control the family‐wise error rate. The ANCOVA analyses were conducted on the subset of participants with complete demographic data (*N* = 374).

Subgroup *t*‐tests comparing instructional conditions within the ADHD subgroup are presented as exploratory analyses given the small sample size (*n* = 32) and should be interpreted with caution.

## 3 Results

### 3.1 Descriptive Statistics

The mean multiple‐choice (MC) score was 80 (SD = 22.8), whereas the constructed‐response (CR) score averaged 68.2 (SD = 27.8), resulting in a total mean score of 72.0 (SD = 20.72). No statistically significant gender differences were identified across all outcome variables. However, female students scored slightly higher on constructed responses (mean 71.46 ± 25.73 vs. 65.28 ± 26.18; *p* = 0.07, Cohen's *d* = 0.13).

### 3.2 Escape Room Versus Traditional Instruction

Students in the escape room group scored significantly lower on CR scores (*d* = 0.28, 95% CI [0.08, 0.48], *p* < 0.01). However, no significant differences were observed in MC (*p* = 0.23) or total scores (*p* = 0.35). These results are presented in Table 1.

**TABLE 1:** Academic characteristics and performance by instructional method.

Variable	Escape room (*n* = 276)	Traditional (*n* = 150)	Statistic	*p*
*Academic characteristics*				
No difficulties, *n* (%)	160 (58.0%)	81 (54.0%)		
Language difficulties, *n* (%)	95 (34.4%)	58 (38.7%)	*χ* ^2^(2) = 0.77	0.68
Learning difficulties, *n* (%)	21 (7.6%)	11 (7.3%)		
*Performance measures*				
MC (%)	81.28 (19.97)	78.22 (27.30)	*t* = 1.21	0.23
CR (%)	64.46 (28.62)	72.06 (25.63)	*t* = 2.80^a^	< 0.01
Total exam score (%)	71.26 (20.34)	73.24 (21.42)	*t* = 0.94	0.35

^a^ Implements Welch's unequal variances test due to rejection of equality of variances assumption (Levene's).

To formally test whether the escape room intervention had differential effects across learner subgroups, two‐way ANCOVAs were conducted with age and gender as covariates. The Escape Room × Learner Status interaction was significant for all three outcomes:

- CR scores: *F*(2, 366) = 3.01, *p* = 0.05, *η*²*p* = 0.02.
- MC scores: *F*(2, 366) = 6.11, *p* < 0.01, *η*²*p* = 0.03.
- Total scores: *F*(2, 366) = 5.45, *p* < 0.01, *η*²*p* = 0.03.

Neither age nor gender was a significant covariate in any model. Holm‐corrected post hoc comparisons revealed distinct patterns across learner subgroups, detailed below.

### 3.3 ADHD/Learning Difficulties (Exploratory)

Students with ADHD/learning difficulties (*n* = 32; ER: *n* = 21, Traditional: *n* = 11) demonstrated higher MC scores under escape room instruction compared to traditional instruction (*M* = 93.33 vs. 70.00; *d* = 0.68, 95% CI [0.17, 1.18]). The ANCOVA simple contrast comparing ER learning difficulties students to the reference group (Traditional, no difficulties) on MC scores was significant (mean difference = +14.60, 95% CI [3.78, 25.42], *p* < 0.01). No significant differences were observed in CR or total scores. Given the small subgroup size, these findings should be considered exploratory and hypothesis‐generating and require replication in adequately powered studies. Performance comparison of students with ADHD by instruction type (Traditional‐ER) is presented in Table 2.

**TABLE 2:** Performance comparison of students with ADHD by instruction type (Traditional‐ER).

Measure	ER (*n* = 21)	Traditional (*n* = 11)	*t*(30)	*p*	95% CI for mean difference	
	*M* (SD)	*M* (SD)			Lower	Upper
MC (%)	92.46 (15.79)	72.73 (27.15)	−2.22^a^	0.04	−38.83	−0.63
CR (%)	72.24 (19.04)	80.91 (17.71)	1.25	0.22	−5.37	22.71
Total scores	79.70 (12.21)	77.49 (13.77)	−0.47	0.64	−12.56	8.13

^a^ Implements Welch's unequal variances test due to rejection of equality of variances assumption (Levene's).

### 3.4 Language Difficulties

Students with language difficulties (*n* = 153; ER: *n* = 95, Traditional: *n* = 58) scored significantly lower under escape room instruction on CR scores (*M* = 54.20 vs. 71.00; *d* = −0.62, 95% CI [−0.96, −0.28]) and total scores (*M* = 61.68 vs. 72.70; *d* = −0.55, 95% CI [−0.88, −0.21]). No significant differences were found in MC scores (*p* = 0.11). The ANCOVA simple contrasts confirmed that the ER disadvantage on CR scores was concentrated among students with language difficulties (mean difference = −15.86, 95% CI [−24.27, −7.45], *p* < 0.01, *d* = −0.63). Holm‐corrected post hoc comparisons showed significant differences between ER language difficulties students and both Traditional language difficulties students (*p* < 0.01) and ER no‐difficulties students (*p* < 0.01). Performance comparison of students with language difficulties by instruction type (Traditional‐ER) is presented in Table 3.

**TABLE 3:** Performance comparison of students with language difficulties by instruction type (Traditional‐ER).

Measure	ER (*n* = 95)	Traditional (*n* = 58)	*t*(151)	*p*	95% CI for mean difference	
	*M* (SD)	*M* (SD)			Lower	Upper
MC (%)	72.89 (20.73)	79.31 (25.99)	1.60^a^	0.11	−1.56	14.39
CR (%)	54.20 (27.40)	71.00 (26.36)	3.73	< 0.01	7.90	25.69
Total scores	61.68 (18.47)	72.70 (22.51)	3.29	< 0.01	4.41	17.64

^a^ Implements Welch's unequal variances test due to rejection of equality of variances assumption (Levene's).

### 3.5 Bloom's Taxonomy Levels

Performance was analysed across different cognitive levels of Bloom's Taxonomy to determine whether the escape room format was differentially associated with lower‐order versus higher‐order thinking skills. Students' scores for CR Levels 1–2 questions were significantly lower in the ER group compared to the traditional instruction group (mean 63.87 ± 28.24 vs. 71.05 ± 24.99; *p* = 0.02, Cohen's *d* = 0.27, 95% CI [0.03, 0.50]). No significant differences were detected for CR Level 3 questions (*p* = 0.10), MC questions at any Bloom's level (*p* = 0.21) or the combined Bloom's Taxonomy 1–2 scores across both formats (*p* = 0.63). These findings should be interpreted with caution given the absence of formal inter‐rater reliability for the Bloom's classification and the unequal distribution of items across cognitive levels.

## 4 Discussion

Prior research has indicated that the utilization of gamification may enhance engagement and interaction among nursing students more effectively than conventional non‐gamified instructional approaches (Grech and Grech 2021). Additionally, these studies have highlighted the potential for integrating gamification techniques into nursing education programs (Sainz‐Rozas et al. 2025). Nonetheless, a research gap persists in the literature, as these inquiries often neglect to address the equitable distribution of these advantages among diverse student populations. The results of this study present a nuanced picture of escape room effectiveness in nursing education, supported by significant interaction effects across all three outcome measures.

The significant Escape Room × Learner Status interactions (CR: *p* = 0.05; MC: *p* < 0.01; Total: *p* < 0.01) formally demonstrate that the escape room intervention was differentially associated with performance across learner subgroups. Although students in the ER group achieved lower scores on CR assessments compared to traditional instruction, this small effect did not translate to overall performance differences. More importantly, the interaction analyses revealed distinct patterns: students with ADHD/learning difficulties showed higher MC performance under ER instruction, whereas students with language difficulties showed lower CR and total scores. These findings challenge the notion that gamified learning approaches offer universal benefits and underscore the importance of considering diverse learner profiles when implementing instructional innovations.

The finding that escape room instruction was associated with lower performance on CR questions but not MC questions is consistent with prior reports of the selective effects of educational gamification (Kim et al. 2024). CR questions necessitate organized thinking and complex written expressions (Hift 2014), which may not be adequately supported in an escape room context. In contrast, the interactive, fast‐paced nature of escape rooms may align more closely with the rapid recognition and decision‐making skills assessed by MC formats. However, contrary to expectations, there were no performance advantages at higher cognitive levels of Bloom's Taxonomy in the ER group, and traditional instruction was associated with significantly better performance on CR questions at lower cognitive levels.

Students with ADHD/learning difficulties (*n* = 32) The escape room instructional approach was differentially associated with academic performance across learners the small subgroup size. This observation is consistent with literature suggesting that gamified media may improve concentration and academic performance for students with ADHD (Kusmawati et al. 2023). The interactive nature of escape rooms may fulfil the need for immediate stimulation and feedback characteristic of ADHD cognitive profiles, with relevance for tasks involving rapid recognition and decision‐making. However, the observation that this pattern did not extend to tasks requiring sustained written expression suggests that escape room instruction may be selectively associated with specific cognitive processes rather than providing comprehensive learning benefits for students with ADHD. These findings warrant replication in adequately powered studies before firm conclusions can be drawn.

The finding concerning students with language difficulties (primarily Hebrew as a non‐native language among Israeli Arab students, *n* = 153) is particularly noteworthy. These students exhibited significantly lower outcomes under escape room instruction, scoring lower CR questions (*d* = −0.63) and total scores (*d* = −0.57). This pattern was confirmed by the significant ANCOVA interaction and Holm‐corrected post hoc comparisons. Notably, performance did not improve even with extra time provided, suggesting that the challenges associated with the escape room format may extend beyond time pressure alone.

Several mechanisms may hypothetically explain these associations, though they were not directly measured in this study and should be considered as hypotheses for future investigation. First, escape rooms typically involve rapid information processing, which may disproportionately burden students already managing language comprehension challenges. Second, complex, multi‐layered instructions common in escape room puzzles may require high‐level language processing that exceeds the capabilities of non‐native speakers, particularly when medical terminology is embedded in narrative contexts. Third, the gamified format may introduce extraneous cognitive load, reducing the cognitive resources available for the already demanding task of processing information in a non‐native language. Fourth, while the escape room was primarily completed individually, the text‐heavy puzzle format may disproportionately disadvantage students less comfortable with reading and interpreting complex instructions in a non‐native language.

These results have implications for educational equity that merit careful consideration. Although escape rooms are widely promoted as engaging pedagogical tools, our findings suggest that they may be differentially associated with outcomes across student populations. The approximately 11‐point gap in total scores observed for students with language difficulties is a substantial difference that could affect academic standing. This is particularly relevant in nursing education, where language‐minoritized students already face significant barriers (Salamonson et al. 2010). However, we note that these findings derive from a single institution and require replication before broader generalizations can be made.

An additional constraint concerns the exams conducted during the study. Due to its retrospective nature, there were differences in the exams held in different academic years, including variations in the format and focus on multiple‐choice (MC) and constructed response (CR) questions among student groups. Although all exams were aligned with the course's educational objectives and the institution's assessment criteria, it was not possible to establish formal psychometric equivalence between the exams in a retrospective analysis. As several of the principal findings in this study were based on differences between MC and CR performance, variation in the composition and weighting of examination items across academic years may have contributed to some of the observed group differences. Consequently, the independent contribution of the instructional approach cannot be fully separated from potential assessment‐related differences. Therefore, these findings should be interpreted with caution and require replication using standardized assessments administered across cohorts.

Our results align with Universal Design for Learning (UDL) principles (CAST 2018), which emphasize the need for multiple means of representation, engagement and expression to accommodate diverse learners. The escape room format, whereas offering novelty and engagement, may inadvertently create barriers by requiring rapid language processing and reducing opportunities for the paced, structured learning that may benefit language learners (Salamonson et al. 2010). Traditional instruction, with its explicit explanations, opportunities for clarification and structured pacing, may provide additional scaffolding that could facilitate learning and knowledge demonstration for some students with language difficulties. However, this interpretation should be considered exploratory and requires confirmation in prospective studies. It is important to note that while academic achievement based on test scores showed differential patterns, the motivational benefits of escape rooms may remain relevant across learner profiles. Increased engagement, motivation, and enhancement of learning environments have been documented in previous research, positioning escape rooms as potential complementary educational tools rather than replacements for traditional methodologies (Manzano‐Leon et al. 2021). Previous research has shown that the immersive setting of escape rooms has the potential to improve critical thinking and problem‐solving skills. However, the present study did not evaluate these specific benefits (Makri et al. 2021). In this context, the choice between escape rooms and traditional instructional methods should be informed by the diversity of student profiles and the educational objectives established by instructors (Pozo‐Sanchez et al. 2022).

### 4.1 Implications for Nursing Education

The findings of this study highlight the importance for nurse educators to carefully consider the assumption that new teaching methods benefit all nursing students equally. Although using digital escape rooms was associated with improved performance on recognition‐based assessments for students with ADHD, it was also linked to decreased performance for students struggling with language skills, especially on tasks that required writing. These findings also reinforce the role of nurse educators in identifying learning barriers early and adapting instructional strategies to support diverse student populations. By continuously evaluating students' responses to innovative teaching approaches, nurse educators can promote equitable learning opportunities while maintaining academic standards and preparing students for safe clinical practice. These results emphasize the need to recognize the diverse learning styles of students when introducing innovative teaching approaches, rather than relying on a one‐size‐fits‐all method. Nurse educators may consider complementing game‐based learning with additional supportive strategies, like providing clear written instructions, promoting self‐reflection, offering guidance on complex assignments, and exploring alternative assessment methods when appropriate. However, since the study focused on a specific group of students, these educational suggestions should be approached cautiously and viewed as preliminary insights until confirmed by future research involving multiple institutions.

## 5 Conclusion

This study provides preliminary evidence that educational innovations may have differential associations with outcomes across learner subgroups. Although the escape room model of instruction was associated with higher MC performance for students with ADHD/learning difficulties on recognition tasks, it was associated with lower CR and total scores for students with language difficulties, with no overall benefit across the full student population. These interaction effects were statistically significant after controlling for age and gender. Our findings suggest that the prevailing narrative that gamification is universally beneficial warrants further scrutiny.

The results raise several points for consideration in nursing education rather than providing definitive recommendations. Given the observational nature of the study and the difficulty in separating the effects of the teaching method from those of the student group, it is recommended to interpret these findings with caution.: escape room‐type instruction may be most effectively implemented as a complementary tool rather than a wholesale replacement for traditional methods. It may be worthwhile investigating whether escape room‐style instruction is particularly beneficial for students with ADHD, especially when the learning objectives emphasize quick recognition and decision‐making skills. The current results indicate that the escape room format may unintentionally create difficulties for students with language challenges. This possibility should be further examined before incorporating it into educational practices, whereas traditional teaching methods may remain an important component of inclusive nursing education, pending confirmation of these findings in future prospective studies.

Future research should explore whether modifying escape room design can improve educational outcomes for students with language challenges. Potential modifications to consider include adding vocabulary support, using multimodal materials, allowing flexible pacing, providing multilingual resources and incorporating structured debriefing sessions. Additionally, prospective studies with randomized designs, larger ADHD subgroups and formal interaction modelling should be conducted to confirm these exploratory findings.

These findings highlight the importance of considering learner diversity when assessing new teaching methods in nursing education. However, it can be difficult to separate the effects of the intervention from factors specific to the group of students being taught. Therefore, these findings highlight the importance of considering the diversity of learners when evaluating educational progress in the field of nursing education. Because the study design was retrospective, non‐randomized, and conducted at a single institution, the results should be viewed as preliminary and approached with caution. It is crucial to replicate these findings in prospective, multi‐center studies before definitive educational guidelines can be developed.

## 6 Limitations

This study has several important limitations that should be considered when interpreting the results.

First, the retrospective quasi‐experimental design with year‐based cohort allocation (2023 = control; 2024–2025 = intervention) limits causal inference. Students were not randomly assigned to conditions, and the year‐based allocation introduces the possibility of cohort effects, including changes in student characteristics, institutional context or unmeasured factors across academic years. Although the groups did not differ significantly on documented learning needs, baseline equivalence was not established for prior academic performance, motivation, digital literacy or other potentially confounding variables.

Second, the exams were not identical across the three academic years. Although all exams assessed the same course learning objectives and curricular content, specific questions differed, and the relative proportions of MC and CR items as well as their weighting in the final grade varied across years. This variation means that observed differences in MC versus CR performance could partly reflect exam design rather than solely instructional effects. This is an important limitation, though it reflects the ecological reality of recurring university courses where exams naturally evolve. Because instructional conditions were completely determined by academic year, the independent effects of the instructional method and examination structure cannot be fully separated in this retrospective design.

Third, the retrospective nature of the study prevented control of intervention fidelity or the measurement of process variables such as time on task, depth of engagement or student perceptions of the learning experience. Although the course instructor confirmed via platform logs that all intervention‐group students accessed and completed the escape room, detailed individual‐level engagement data (e.g., time spent, number of attempts) were not available for analysis. The specific mechanisms underlying the observed associations for language‐minoritized students remain hypothetical without qualitative data.

Fourth, the study was conducted at a single institution with one instructor, which limits generalizability. The specific escape room design, implementation approach and instructor characteristics may have influenced outcomes. Different implementations might yield different results.

Fifth, the sample size for the ADHD/learning difficulties subgroup was small (*n* = 32; ER: *n* = 21, Traditional: *n* = 11), limiting statistical power and the stability of effect estimates. The ADHD‐specific findings should be treated as exploratory and hypothesis‐generating. Moreover, the analysis grouped ADHD and learning difficulties together as a single institutional subgroup instead of treating them as separate conditions. As a result, the observed correlations cannot be solely attributed to either ADHD or learning difficulties. It is recommended that future research efforts focus on investigating these populations independently whenever possible.

Sixth, 53 students (12.3%) had missing demographic data due to institutional data retention policies. Although these cases were distributed across all three academic years and the missingness appears to be related to administrative processes, non‐random missingness cannot be fully excluded. The ANCOVA analyses were conducted on the subset with complete demographic data (*N* = 374), and findings should be interpreted in light of this reduced sample.

Seventh, the Bloom's Taxonomy classification of exam items was performed by the study authors for the purpose of this study. Formal inter‐rater reliability testing was not conducted, and only cognitive levels 1–3 were identified. Conclusions regarding Bloom's Taxonomy levels should be considered exploratory.

Eighth, we did not measure long‐term retention, transfer of knowledge to clinical settings or professional competency development. The focus on cognitive outcomes also does not capture potential socio‐emotional effects, which may be particularly relevant for already‐marginalized student populations.

Finally, the ANCOVA analyses, whereas providing formal interaction tests, included age and gender as covariates but could not adjust for all potential confounders. The observed associations should not be interpreted as causal effects of the escape room intervention.

## Author Contributions

**Dorit Weil‐Lotan:** conceptualization, writing – review and editing, methodology. **Revital Harari:** data curation, writing – review and editing, investigation, formal analysis. **Carolina Tannenbaum‐Baruchi:** investigation, data curation, visualization. **Miriam Theilla:** conceptualization, writing – original draft, methodology, formal analysis, supervision, visualization, writing – review and editing.

## Funding

The authors have nothing to report.

## Ethics Statement

Ethical approval was obtained from the relevant institutional review board to access academic data within the institutional framework. Approved by the Institutional Ethics Committee of The Tel Aviv Yaffo Academic College (No. 005‐24‐MTA). The study was conducted in accordance with the ethical principles of the Declaration of Helsinki and relevant institutional and national research ethics guidelines.

## Consent

The research involved a retrospective analysis of data extracted from an academic learning management system. All data were fully anonymized prior to analysis to ensure the protection of individual privacy and confidentiality. Due to the retrospective, non‐interventional nature of the study and the use of anonymized data, the requirement for informed consent was waived by the ethics committee.

## Conflicts of Interest

The authors declare no conflicts of interest.

## Supporting information


**Appendix S1:** Examples of virtual escape room tasks used in the nursing theory course.

## Data Availability

The data that support the findings of this study are available from the corresponding author upon reasonable request.

## References

[nop270882-bib-0001] Asad, M. M. , A. Naz , P. Churi , and M. M. Tahanzadeh . 2021. “Virtual Reality as Pedagogical Tool to Enhance Experiential Learning: A Systematic Literature Review.” Education Research International 2021, no. 1: 7061623.

[nop270882-bib-0002] Borrego, C. , C. Fernandez , I. B. Garcia , and S. Robles . 2017. “Room Escape at Class: Escape Games Activities to Facilitate the Motivation and Learning in Computer Science.” Journal of Technology and Science Education 7, no. 2: 162–171.

[nop270882-bib-0003] Casey, K. , R. Fink , C. Jaynes , L. Campbell , P. Cook , and V. Wilson . 2011. “Readiness for Practice: The Senior Practicum Experience.” Journal of Nursing Education 50, no. 11: 646–652. 10.3928/01484834-20110817-03 21846072

[nop270882-bib-0004] CAST . 2018. “Universal Design for Learning Guidelines Version 2.2.” http://udlguidelines.cast.org.

[nop270882-bib-0005] Corlett, J. , J. W. Palfreyman , H. J. Staines , and H. Marr . 2003. “Factors Influencing Theoretical Knowledge and Practical Skill Acquisition in Student Nurses: An Empirical Experiment.” Nurse Education Today 23, no. 3: 183–190. 10.1016/s0260-6917(02)00232-0 12672386

[nop270882-bib-0006] Crawford, T. , and S. Candlin . 2013. “A Literature Review of the Language Needs of Nursing Students Who Have English as a Second/Other Language and the Effectiveness of English Language Support Programmes.” Nurse Education in Practice 13, no. 3: 181–185. 10.1016/j.nepr.2012.09.008 23041163

[nop270882-bib-0007] Del Prato, D. , E. Bankert , P. Grust , and J. Joseph . 2011. “Transforming Nursing Education: A Review of Stressors and Strategies That Support Students' Professional Socialization.” Advances in Medical Education and Practice 2: 109–116. 10.2147/AMEP.S18359 PMC3661250 23745082

[nop270882-bib-0008] DuPaul, G. J. , R. A. Ervin , C. L. Hook , and K. E. McGoey . 1998. “Peer Tutoring for Children With Attention Deficit Hyperactivity Disorder: Effects on Classroom Behavior and Academic Performance.” Journal of Applied Behavior Analysis 31, no. 4: 579–592. 10.1901/jaba.1998.31-579 PMC1284149 9891395

[nop270882-bib-0009] Ghasemi, M. R. , H. K. Moonaghi , and A. Heydari . 2020. “Strategies for Sustaining and Enhancing Nursing Students' Engagement in Academic and Clinical Settings: A Narrative Review.” Korean Journal of Medical Education 32, no. 2: 103–117. 10.3946/kjme.2020.159 PMC7272374 32486620

[nop270882-bib-0010] Gormley, M. J. , T. Pinho , B. Pollack , et al. 2018. “Impact of Study Skills and Parent Education on First‐Year GPA Among College Students With and Without ADHD: A Moderated Mediation Model.” Journal of Attention Disorders 22, no. 4: 334–348. 10.1177/1087054715594422 PMC4715995 26187415

[nop270882-bib-0011] Grech, J. , and J. Grech . 2021. “Nursing Students' Evaluation of a Gamified Public Health Educational Webinar: A Comparative Pilot Study.” Nursing Open 8, no. 4: 1812–1821. 10.1002/nop2.826 PMC8186683 33675289

[nop270882-bib-0012] Guckian, J. , L. Eveson , and H. May . 2020. “The Great Escape? The Rise of the Escape Room in Medical Education.” Future Healthcare Journal 7, no. 2: 112–115. 10.7861/fhj.2020-0032 PMC7296573 32550277

[nop270882-bib-0013] Hift, R. J. 2014. “Should Essays and Other ‘Open‐Ended’‐Type Questions Retain a Place in Written Summative Assessment in Clinical Medicine?” BMC Medical Education 14: 249. 10.1186/s12909-014-0249-2 PMC4275935 25431359

[nop270882-bib-0014] Hoeve, Y. T. , G. Jansen , and P. Roodbol . 2014. “The Nursing Profession: Public Image, Self‐Concept and Professional Identity. A Discussion Paper.” Journal of Advanced Nursing 70, no. 2: 295–309. 10.1111/jan.12177 23711235

[nop270882-bib-0015] Kallivayalil, R. A. 2012. “The Importance of Psychiatry in Undergraduate Medical Education in India.” Indian Journal of Psychiatry 54, no. 3: 208–216. 10.4103/0019-5545.102336 PMC3512355 23226842

[nop270882-bib-0016] Kim, C. , H. Na , N. Zhang , and C. Bai . 2024. “Escape Rooms for Education: A Meta‐Analysis.” International Journal of Instruction 17, no. 4: 219–234.

[nop270882-bib-0017] Kooij, J. J. S. , D. Bijlenga , L. Salerno , et al. 2019. “Updated European Consensus Statement on Diagnosis and Treatment of Adult ADHD.” European Psychiatry 56: 14–34. 10.1016/j.eurpsy.2018.11.001 30453134

[nop270882-bib-0018] Kusmawati, A. P. , F. Fahrurrozi , and A. Supena . 2023. “Increasing Concentration of Attention Deficit Hyperactivity Disorder (ADHD) Students Through Gamification Learning Media in Indonesian Inclusion Elementary School.” International Journal of Special Education 38, no. 1: 169–184.

[nop270882-bib-0019] Lavoie‐Tremblay, M. , L. Sanzone , T. Aubé , and M. Paquet . 2022. “Sources of Stress and Coping Strategies Among Undergraduate Nursing Students Across All Years.” Canadian Journal of Nursing Research 54, no. 3: 261–271. 10.1177/08445621211028076 PMC9379378 34192949

[nop270882-bib-0020] Legaki, N. Z. , N. Xi , J. Hamari , K. Karpouzis , and V. Assimakopoulos . 2020. “The Effect of Challenge‐Based Gamification on Learning: An Experiment in the Context of Statistics Education.” International Journal of Human‐Computer Studies 144: 102496. 10.1016/j.ijhcs.2020.102496 PMC7293851 32565668

[nop270882-bib-0021] Liepinaitiene, A. , V. Jotautis , S. Jankauskaite , et al. 2024. “Prevalence and Associations of Neurodevelopmental and Mental Health Disorders With Academic and Well‐Being Challenges Among Nursing and Midwifery Students: A Cross‐Sectional Study.” European Journal of Midwifery 8: 74. 10.18332/ejm/195808. PMC11635650 39670193

[nop270882-bib-0022] Makri, A. , D. Vlachopoulos , and R. A. Martina . 2021. “Digital Escape Rooms as Innovative Pedagogical Tools in Education: A Systematic Literature Review.” Sustainability 13, no. 8: 4587.

[nop270882-bib-0023] Manzano‐Leon, A. , P. Camacho‐Lazarraga , M. A. Guerrero , et al. 2021. “Between Level Up and Game Over: A Systematic Literature Review of Gamification in Education.” Sustainability 13, no. 4: 2247.

[nop270882-bib-0024] Pozo‐Sanchez, S. , G. Lampropoulos , and J. Lopez‐Belmonte . 2022. “Comparing Gamification Models in Higher Education Using Face‐to‐Face and Virtual Escape Rooms.” Journal of New Approaches in Educational Research 11, no. 2: 307–322.

[nop270882-bib-0025] Sainz‐Rozas, P. B. , E. Balaguer‐Lopez , P. Garcia‐Molina , and P. G. Martinez . 2025. “Evaluation of Paediatric Nursing Students' Medication Calculation Competence: An Observational Study Using a Gamified Contest.” Nursing Open 12, no. 9: e70295. 10.1002/nop2.70295 PMC12461758 40996097

[nop270882-bib-0026] Salamonson, Y. , J. Koch , R. Weaver , B. Everett , and D. Jackson . 2010. “Embedded Academic Writing Support for Nursing Students With English as a Second Language.” Journal of Advanced Nursing 66, no. 2: 413–421. 10.1111/j.1365-2648.2009.05158.x 20423424

[nop270882-bib-0027] Su, W. M. , and P. J. Osisek . 2011. “The Revised Bloom's Taxonomy: Implications for Educating Nurses.” Journal of Continuing Education in Nursing 42, no. 7: 321–327. 10.3928/00220124-20110621-05 21707023

[nop270882-bib-0028] Ulrich, B. , C. Krozek , S. Early , C. H. Ashlock , L. M. Africa , and M. L. Carman . 2010. “Improving Retention, Confidence, and Competence of New Graduate Nurses: Results From a 10‐Year Longitudinal Database.” Nursing Economics 28, no. 6: 363–375. 21291057

[nop270882-bib-0029] Vabo, G. , Å. Slettebø , and M. Fossum . 2022. “Nursing Students' Professional Identity Development: An Integrative Review.” Nordic Journal of Nursing Research 42, no. 2: 62–75.

[nop270882-bib-0030] World Health Organisation (WHO) . 2019. International Classification of Diseases for Mortality and Morbidity Statistics. 11th ed. World Health Organisation. https://icd.who.int/.

[nop270882-bib-0031] Zhao, X. , W. K. Zheng , X. H. Wang , et al. 2024. “Influence of Perceived Stress on Professional Identity Among Nursing Students: A Chain Mediating Role of Self‐Control and Self‐Directed Learning Ability.” Frontiers in Medicine 11: 1429014. 10.3389/fmed.2024.1429014 PMC11588480 39600932

