# Supplementary material for: One Size Does not Fit All: Gamified Escape Room Learning for Students With ADHD and Language Difficulties in a Quasi‐Experimental Cohort Study
Source: Nurs Open. 2026 Sep 30;13(10):e70882. doi: 10.1002/nop2.70882 (PMC13625671; doi:10.1002/nop2.70882)
Supplement: Supplementary file 1 [file NOP2-13-e70882-s001.docx]

**Supplementary Material S1. Examples of Virtual Escape Room Tasks Used in the Nursing Theory Course**

|  | **Examples of questions**   1. What is the conceptualization of suffering within Florence Nightingale's Environmental Theory? 2. According to Maslow's hierarchy of needs, which nursing intervention should take priority for a patient admitted to a hospital? 3. Match the nursing metaparadigm concepts with their corresponding definitions within the framework of various nursing theories in a drag-and-drop exercise. |
| --- | --- |
